# Harnessing the Potential of Hollow Graphitic Carbon Nanocages for Enhanced Methanol Oxidation Using PtRu Nanoparticles

**DOI:** 10.3390/polym16192684

**Published:** 2024-09-24

**Authors:** Zatil Amali Che Ramli, Jagadeesh Pasupuleti, Siti Kartom Kamarudin, Azran Mohd Zainoodin, Wan Nor Roslam Wan Isahak, S. P. Koh, Sieh Tiong Kiong

**Affiliations:** 1 Institute of Sustainable Energy (ISE), Universiti Tenaga Nasional (UNITEN), Putrajaya Campus, Jalan IKRAM-UNITEN, Kajang 43000, Selangor, Malaysia; zatilamali@yahoo.com (Z.A.C.R.); siehkiong@uniten.edu.my (S.T.K.); 2 Fuel Cell Institute, Universiti Kebangsaan Malaysia, Bangi 43600, Selangor, Malaysia; ctie@ukm.edu.my (S.K.K.); azrans@ukm.edu.my (A.M.Z.); 3 Department of Chemical and Process Engineering, Faculty of Engineering and Built Environment, Universiti Kebangsaan Malaysia, Bangi 43600, Selangor, Malaysia; wannorroslam@ukm.edu.my

**Keywords:** DMFC, MOR, anodic catalyst, PtRu nanoparticles, carbon nanocages (CNC) support, polypyrrole-CNC, energy production

## Abstract

Direct Methanol Fuel Cell (DMFC) is a powerful system for generating electrical energy for various applications. However, there are several limitations that hinder the commercialization of DMFCs, such as the expense of platinum (Pt) at market price, sluggish methanol oxidation reaction (MOR) due to carbon monoxide (CO) formation, and slow electrooxidation kinetics. This work introduces carbon nanocages (CNCs) that were obtained through the pyrolysis of polypyrrole (Ppy) as the carbon source. The CNCs were characterized using BET, XRD, HRTEM, TEM, SEM, and FTIR techniques. The CNCs derived from the Ppy source, pyrolyzed at 750 °C, exhibited the best morphologies with a high specific surface area of 416 m^2^g^−1^, allowing for good metal dispersion. Subsequently, PtRu catalyst was doped onto the CNC-Ppy750 support using chemical reduction and microwave-assisted methods. In electrochemical tests, the PtRu/CNC-Ppy750 electrocatalyst demonstrated improved CO tolerance and higher performance in MOR compared to PtRu-supported commercial carbon black (CB), with values of 427 mA mg^−1^ and 248 mA mg^−1^, respectively. The superior MOR performance of PtRu/CNC-Ppy750 was attributed to its high surface area of CNC support, uniform dispersion of PtRu catalyst, and small PtRu nanoparticles on the CNC. In DMFC single-cell tests, the PtRu/CNC-Ppy750 exhibited higher performance, approximately 1.7 times higher than PtRu/CB. In conclusion, the PtRu/CNC-PPy750 represents a promising electrocatalyst candidate for MOR and anodic DMFC applications.

## 1. Introduction

One of the United Nations Sustainable Development Goals (SDG 7) is “affordable and clean energy”. To reach this goal, several policies and regulatory measures are needed, including the investment in renewable energy infrastructure, the establishment of feed-in tariffs and other financial incentives, and the development of energy efficiency standards and labeling schemes. The goal is to ensure universal access to modern energy services, such as electricity and clean cooking fuels, while simultaneously boosting the use of renewable energy sources and enhancing energy efficiency for all by 2030. In accordance with the United Nations’ Sustainable Development Goals (SDG 7)’s objective and directions, the development of technologies should be efficient, user-friendly, and capable of satisfying the present energy requirements.

Due to the ever-increasing energy demand and environmental pollution, numerous research studies have been focused to the clean and green technologies. Direct methanol fuel cell (DMFC) has attracted intense attention as a powerful alternative energy source for various applications, including portable devices and stationary applications. This is due to its advantages, such as easy fuel storage, wide availability, high energy efficiency, and greenhouse gas emission reduction [1,2,3]. Compared with other fuel cells, the DMFC offers several advantages, including low operating temperature, simple design, relatively low cost, and ease of handling, making it the most popular type of Direct Alcohol Fuel Cell (DLFC) [4,5,6,7].

To date, Pt has been considered the most effective electrocatalyst for DMFCs due to its high catalytic activity in the methanol oxidation reaction (MOR) [8]. However, the use of Pt catalyst in commercial DMFC fuel cell systems faces several problematic issues, such as the high market price of Pt, the formation of carbon monoxide (CO) intermediates, and slow methanol oxidation rates [9,10]. To address the issue of CO formation, it is necessary to modify Pt catalyst by incorporating a second metal (e.g., Sn, Mo, Co, Ru, Fe) or alloying it with other materials that can overcome these issues [11]. Among various bimetallic platinum catalysts, PtRu was considered as the best bimetallic electrocatalyst for MOR. This is because the incorporation of Ru as a second metal promotes the formation of CO_2_ from CO intermediates at a lower potential energy [12,13,14]. Unfortunately, it is still very challenging to maintain or improve the catalytic activity and durability in MOR. Considerable effort has been devoted to addressing these issues in the MOR by reducing the Pt usage by various methods, including the incorporation of carbon support materials.

Various nanostructured carbon supports have been used for Pt-based catalysts, including carbon nanotubes [15,16], carbon nanofibers [17,18], graphene [19,20], multiwall carbon nanotubes [21,22], mesoporous carbons [23,24,25], and other carbon materials. These carbon supports offer advantages over Vulcan XC-72, such as good adsorption, low density, high surface area, excellent electrical conductivity, and high catalytic/electrocatalytic activity. The main purpose of using a support material is to increase the specific surface area of the Pt nanoparticles. Additionally, carbon supports act as an important medium to avoid particle agglomeration during the dispersion of the catalyst metal particles, reduce carbon corrosion problem, and reduce the Pt loading as the main catalyst [26,27,28]. For instance, Zhang et al. [29] successfully deposited Pt nanoparticles on 3D hierarchically porous graphitic carbon nitride. The high specific surface area of the support resulted in highly dispersed Pt nanoparticles and enhanced methanol electrooxidation performance. Eshghi et al. [30] reported that Pt and Pt-Fe nanoparticles are uniformly dispersed on reduced graphene oxide and exhibited higher electrocatalytic activity in MOR and improved durability compared to the commercial Pt/C electrocatalyst. Furthermore, Sieben et al. [31] employed SWCNT as PtRu catalyst support, and demonstrated its strong influence for dispersion of catalyst, catalyst particle size and metal loading. Meanwhile, Wang et al. [32] combined the graphene sheets and CNT support, which showed a 3D porous structure providing a large surface area for the even dispersion of PtRu nanoparticles, thereby enhancing the electrocatalytic performances in MOR. These previous research studies indicated the potential of each carbon support as an electrocatalyst supporting material and their extensive use in MOR and DMFC. Therefore, a new carbon support, carbon nanocages (CNC), has been introduced in this present work due to its potential for possessing a high surface area, strong corrosion resistance, and cost-effective production, making it suitable for dispersing active catalysts such as PtRu.

**Figure 1:**
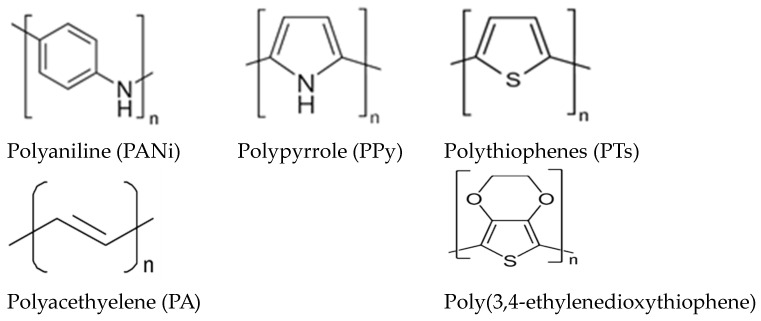
Several types of conductive polymer [41].

The use of carbon nanocages (CNCs) as a carbon support material offers several advantages, primarily due to their very high specific surface area and high electrical conductivity [33,34]. Generally, CNCs have been used in various applications such as catalyst supports [34], adsorbents [35,36], lithium-ion batteries [37], and supercapacitors [38]. Over time, a variety of synthesis methodologies have been developed for the preparation of carbon nanocages (CNCs). In most synthesis methods, acetylene, ethanol, and pyridine are commonly used as carbon sources in the pyrolysis technique [39]. In this present work, we introduce the synthesis of carbon nanocages as the support material from two types of conductive polymer namely polyaniline (PANi) and polypyrrole (Ppy), as the CNCs source through the pyrolysis technique. Meanwhile, the addition of bimetallic of Ru is aimed to reduce the amount of Pt as main catalyst and improving the CO tolerance during MOR process. PANiPpy contain a nitrogen element in their ring structure, as shown in Figure 1. Previous studies by Jiang et al. [34] have reported that nitrogen functionalization of carbon materials contributes to better catalyst morphology, enhanced catalytic performance, and improved durability of the catalyst. Moreover, nitrogen-doped carbon support also facilitates the better dispersion of the nano catalyst over carbon support and prevents particle agglomeration [34,40].

The bimetallic PtRu supported carbon nanocages (CNCs) support was synthesized microwave-assisted reduction method and their catalytic performance was evaluated through methanol oxidation reaction. The microwave-assisted technique is applied in this presents study as it provides the necessary heat for the reduction in metals from its precursors in very short period [42,43]. Furthermore, the catalytic activities of PtRu/CNC-Ppy750 in MOR were also compared with other PtRu-based catalysts and commercial PtRu/CBs. Results from cyclic voltammetry (CV) and chronoamperometry (CA) tests demonstrated that PtRu/CNC-Ppy750 electrocatalyst exhibited highly effective catalytic activity, high CO tolerance, superior durability, and great potential as an electrocatalyst for future DMFC technology. These results highlight the synergetic interaction between the PtRu catalyst and the CNCs supporting material. Therefore, the new CNC supporting material with the PtRu catalyst holds great promise as an electrocatalyst for future DMFC applications.

## 2. Materials and Methods

### 2.1. Materials and Chemicals

Pt precursor (H_2_PtCl_6_) bought from Merck company (Darmstadt, Germany). Ru precursor (RuCl4), polypyrrole solution (99% purity), aniline hydrochloride (C_6_H_5_NH_2_·HCl, 99.95%), magnesium sulfate heptahydrate (MgSO_4_·7H_2_O) as an MgO source, ammonium peroxydisulfate as an oxidant, isopropyl alcohol as solvent, nitric acid (HNO_3_), ethylene glycol (EG), sulfuric acid (H_2_SO_4_), nafion solution, methanol solution (CH_3_OH), and Nafion-117 membrane were bought from Sigma Aldrich company (St. Louis, MO, USA). Vulcan XC72 carbon black was purchased from Fisher Scientific company (Waltham, MA, USA). Pt black (HiSPEC 1000) was purchased from Alfa Easer, Ward Hill, MA, USA.

### 2.2. Structural Characterizations

The functionality of the groups in the samples were determined using their FTIR spectra. The morphology and structure of the samples were determined using a field emission scanning electron microscope (FESEM, ZEISS Supra VP55), transmission electron microscope (TEM, JEOL JEM-2100), and a high-resolution transmission electron microscope (HRTEM) operated at 200 kV. The specific surface area, pore size, pore volume, and pore diameter of the samples were determined by the Brunauer–Emmet–Teller (BET) method using a nitrogen adsorption instrument (Micrometics ASAP 2010). The samples were degassed at 100 °C for 24 h prior to the analysis. The pore size distribution was calculated from the adsorption–desorption of the isotherm using the Barret–Joyner–Halenda (BJH) model, while the crystallinity of the samples was analyzed using a Brucker DB-advance X-ray Diffractometer (XRD). The analyses were performed using the Cu kα radiation at 2θ ranging from 10° to 80° for a 1-g sample. Scherer’s equation was employed to calculate the estimated crystallite size of the catalyst as following Equation (1) [1]:

d = Kλ/*B* cos θ (1)

where,

d = estimated crystallite size,
K = is a dimensionless shape factor, with a value close to unity. The shape factor has a typical value of about 0.94,
λ = X-ray wavelength (constant value of 0.154)
*B* = line broadening at half the maximum intensity (FWHM), after subtracting the instrumental line broadening (in radians), and
θ = Bragg’s angle.

### 2.3. Preparation of Hollow Carbon Nanocages (CNCs) from Polypyrrole (PPy) and Polyaniline (PANI)

In this work, polyaniline (PANi) and polypyrrole (Ppy) polymers were used as the carbon source for CNCs that were prepared using a modified chemical oxidative method [44]. The modified chemical oxidative method is relatively simple and straightforward compared to other polymerization techniques. It involves the oxidative polymerization of the monomer in the presence of an oxidizing agent, such as ammonium persulfate (APS). The simplicity of the process makes it accessible to researchers and facilitates its use in various applications. Moreover, this method generally provides high polymerization yields, resulting in a large amount of polymer being synthesized. This is advantageous when large quantities of PPy are required for applications in fields such as energy storage, sensing, and electronic devices. In addition, by adjusting various reaction parameters, such as the concentration of reactants, reaction time, temperature, and choice of oxidizing agent, it is possible to influence the morphology, molecular weight, and conductivity of the synthesized polymer. To prepare PANi as carbon source, firstly 259 mg aniline hydrochloride was added to 5 mL deionized water and was stirred for 10 min at a temperature of 60 °C [44]. Meanwhile, 571 mg ammonium peroxydisulfate (APS) was mixed in 5 mL deionized water and stirred for 10 min. The prepared APS solution was then added to the mixture of aniline with continuous stirring for an additional 10 min to complete the polymerization process. Finally, magnesium sulfate heptahydrate as a MgO template for the carbon cage was added and stirred vigorously for 1 h. During the polymerization process (PANi or PPy), the MgO used as the CNC template was mixed and stirred for 1 h. Subsequently, carbon nanocages (CNCs) were obtained from the PANi source through pyrolysis under nitrogen gas flows at the temperatures of 600 °C, 750 °C, and 900 °C in a horizontal quartz furnace. The furnace was heated to the target temperature, and a N_2_ gas flow rate of 5°/min was initiated through the glass tube prior to the furnace. After 2 h of reaction, the as-grown samples were cooled at room temperature and collected. The carbon materials containing the MgO templates were then treated by the acid leaching process in 1 M HNO_3_ for 6 h in order to remove the MgO templates. Finally, the carbon samples were washed several times with absolute ethanol and deionized water until neutral pH was reached. Finally, the CNCs sample was dried in a vacuum oven at 100 °C for 6 h. The CNCs from the Ppy source were denoted as CNC-Ppy600, CNC-Ppy750, and CNC-Ppy900 for operation at the temperatures of 600 °C, 750 °C and 900 °C, respectively. The experiments were repeated using Ppy as the carbon source for CNCs with the same method and temperatures as described above.

### 2.4. Preparation of CNCs Supported PtRu Nanoparticles (PtRu/CNC) and Commercial Carbon Black Supported PtRu Nanoparticles (PtRu/CB)

The PtRu catalyst supported on CNCs was prepared by the alcohol-reduction method in which ethylene glycol (EG) is used as the solvent and reducing agent. There are some advantageous of choosing alcohol reduction method:

(i) The alcohol reduction methods is relatively simple and the equipment/reagents needed are readily available. Normally, it operates under mild reaction conditions, including ambient temperature and pressure. This mildness is advantageous as it simplifies the experimental setup and reduces the energy requirements for synthesis.
(ii) It is environmentally friendly compared to other synthesis methods. Alcohol solvents, such as ethylene glycol or isopropanol, are relatively non-toxic and reduce the generation of hazardous waste.
(iii) It tends to produce high purity of PtRu nanoparticles with good crystallinity. The alcohol solvents helps in reducing impurities and preventing the formation of unwanted byproducts. The resulting PtRu nanoparticles exhibit well-defined crystal structures, which can enhance their electrocatalytic properties.
(iv) It promotes the formation of homogeneous and uniform distribution of Pt and Ru atoms over supporting material. This is attributed to the reduction mechanism and the coordination ability of alcohol molecules, which facilitate the mixing of Pt and Ru at the atomic level. The resulting homogeneous structure strongly influences the catalytic performance and stability of the PtRu catalyst.

These factors make them advantageous for synthesizing bimetallic PtRu compared to other methods.

Typically, CNCs are first sonicated in an EG solution first. The EG was prepared in the volume ratio of 70:30 (EG: DI water). Chloroplatinic acid (Pt source), and ruthenium chloride (Ru source) precursors were then prepared separately by dissolving in EG solutions and sonicating for 15 min. Next, both mixtures were added into the carbon slurry. The mixture was heated three times using microwave irradiation, with 2 min on and 1 min off intervals. The desired nominal amount of Pt and Ru, set at 20 wt.% (weight percentage), was deposited. After cooling to room temperature, 3 M acid hydrochloric was added (until pH 1) to the suspension containing the PtRu catalyst and CNC support to induce the sedimentation of the as-synthesized sample. After that step, the catalyst was filtered and washed with ethanol and deionized water several times to remove the chloride ions. The prepared samples were denoted as PtRu/CNC-Ppy750, for the catalyst supported on CNC from the Ppy source at the temperature of 750 °C. Finally, the prepared samples underwent physical and chemical characterization, half-cell testing for electrochemical performance, and were later used for testing the DMFC performance. The similar procedure was used for the preparation of PtRu/CB for comparison with the PtRu/CNC electrocatalyst.

### 2.5. Electrochemical Performance of PtRu Electrocatalysts

To investigate the electrochemical performance of the synthesized CNCs as a catalyst support for the DMFC electrode, the CNC materials synthesized at 750 °C (which is the temperature at which the optimal carbon cage shape was obtained) were studied further. An electrochemical study was conducted on the PtRu catalyst (20% catalyst deposited) supported on the synthesized CNC material (PtRu/CNC-Ppy750). For comparison, a catalyst with the same amounts of Pt and Ru deposited on a commercial carbon black was also synthesized (PtRu/CB).

All electrochemical tests were carried out to examine the activities of the electrocatalysts and their stability towards MOR. The CV test was carried out in a 0.5 M H_2_SO_4_ electrolyte and a 2.0 M CH_3_OH solution, with the potential cycling from 0 to 1.0 V at a scan rate of 50 mV s^−1^. The Pt gauze electrode was used as the counter electrode, and an Ag/AgCl electrode was used as the reference electrode during MOR. Meanwhile, the working electrode consisting of a thin layer of the synthesized electrocatalyst was prepared by the deposition of a catalyst ink on a 0.0707 cm^2^ glassy carbon disk according to the method described in previous studies [45]. The electrocatalyst loading on the working electrode is 0.2 cm^−2^. The electrolyte was purged with saturated N_2_ gas flow at room temperature for 30 min for CV and CA analyses. The CA curves were recorded for 3600 s at a fixed voltage of 0.4 V in a 0.5 M H_2_SO_4_ electrolyte and a 2.0 M CH_3_OH solution. All obtained data were analyzed. For further investigation for the stability of the prepared electrocatalyst, the retention value of each the electrocatalyst is evaluated by CA analysis, which focuses on the level of resistance or force of the electrocatalyst for a certain period as Equation (2) [46];

(2) $$Retention\;value\left(\%\right)=\frac{Final\;current\;density}{Initial\;current\;density}\times 100\%$$

### 2.6. Preparation of Membrane Electrode Assembly (MEA)

For the membrane electrode assembly (MEA), PtRu/CNC-ppy750 was used as the anode catalyst layer, while Pt black (HiSPEC 1000, Alfa Easer, USA) was used for the cathode catalyst layer with 2 mg/cm^2^ catalyst loading for each electrode. Nafion-117 membrane was used as the electrolyte membrane. The Nafion-117 membrane was sandwiched between the anode and the cathode layer by hot-pressing at 135 °C and 50 kPa for 3 min [47]. The same procedure was used for the fabrication of MEA using PtRu/CB as the anode catalyst layer.

### 2.7. DMFC Single Cell Test

The performance of a single cell was evaluated in a passive single cell with an active area of 4 cm^2^. Air was supplied directly through the opening area of the cathode end plate while a methanol tank was built in the anode fixture. The passive DMFC was fueled with dilute methanol (10 mL) and the testing was conducted at room temperature. The polarization curves of the passive DMFC were measured in the Potentiostat/Galvanostat mode using a WonATech instrument. Prior to the performance testing, the MEA were activated for 12 h with a 2.0 M methanol solution. The MEA with the anode catalyst layer was further tested with a 2.0 M methanol solution to study their performance in the DMFC and the results were presented using the polarization curve and long-term stability data.

## 3. Results and Discussion

### 3.1. Structural Characterization of CNC Supports and Their Electrocatalysts

**Figure 2:**
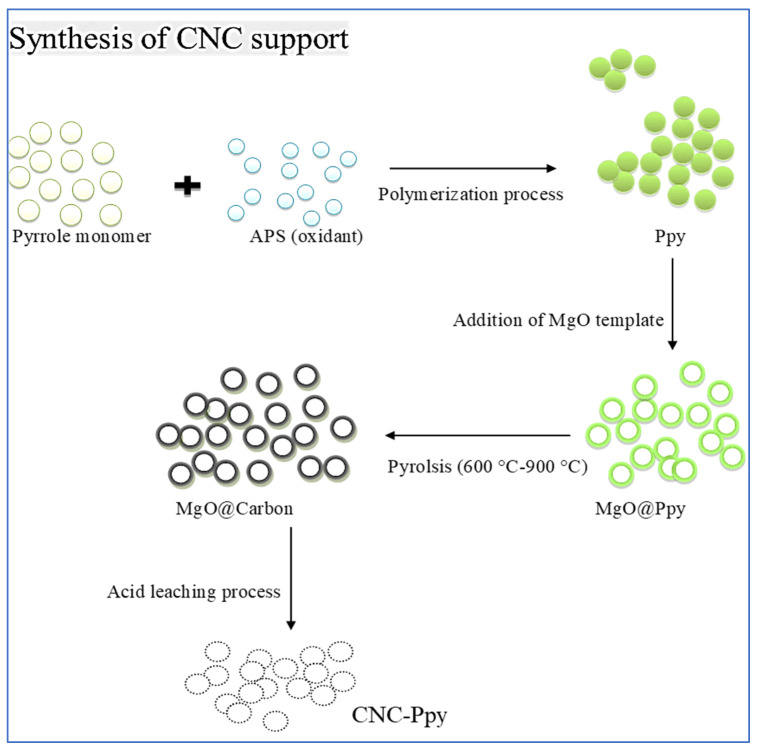
Schematic diagram for carbon nanocages synthesis starting from PPy as a CNC source.

As shown in the schematic illustration in Figure 2, the synthesis of hollow carbon nanocages (CNCs) involves a simple route. The first step involves the polymerization process to prepare the polymer as carbon source, using the same method employed by Amali and colleagues [44]. Then, the polymer (e.g., polypyrrole) was mixed with magnesium oxide (MgO) as the cage template. The experiment suggests that the growth mechanism of the resulting carbon cage involves carbon adsorption on the metal template, followed by the formation of a graphitic layer as a cap during the carbonization process at a specific temperature. During the acid leaching process, the cap then lifts off the MgO metal template, producing a hollow graphitic carbon structure called the carbon nanocage (CNC).

In detail, the synthesis of hollow graphitic CNCs involves several steps. First, the polymer (e.g., polypyrrole) undergoes polymerization using its oxidant, APS, followed by the addition of a magnesium template to produce the MgO@Ppy particles. Carbonization is then performed through pyrolysis of MgCO@Ppy for at 600 °C, 750 °C, and 900 °C for 2 h to produce graphitic CNCs-coated MgO particles (MgO@CNCs). Finally, an acid leaching process is carried out to isolate the MgO and carbon, resulting in the production of hollow graphitic CNCs.

**Figure 3:**
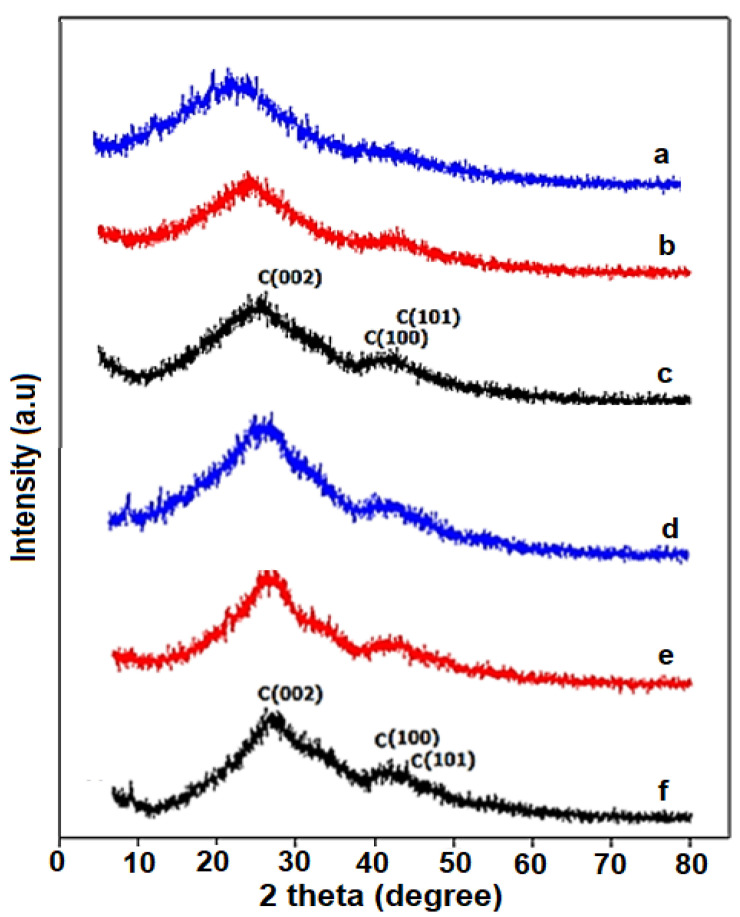
XRD for carbon sample (**a**) CNC-PANi600, (**b**) CNC-PANi750, (**c**) CNC-PANi900, (**d**) CNC-PPy600, (**e**) CNC-Ppy750, (**f**) CNC-Ppy900.

X-ray diffraction analysis (XRD) is an important characterization method for the investigation of the nature of the prepared carbon materials. Figure 3f illustrates the XRD patterns of the carbon material from PPy and PANi sources at various temperatures (600 °C, 750 °C, and 900 °C). The XRD diffraction results indicated that all carbonaceous samples prepared from this simple pyrolysis route show graphitic carbon structure (JCPDS file: 03-065-6212). All the samples clearly exhibit two main diffraction peaks located at 2 theta of approximately 26° and 42° [38]. The peak at 26° corresponds to the (002) plane, while the peak at 42° corresponds to the (100) or (101) planes, indicating that the carbon materials are in the graphite form. A strong crystalline peak of the (002) plane is clearly observed for the highest temperature (900 °C) sample for CNC-Ppy900 (Figure 3f) from the Ppy source, indicating better graphitic structure compared to the samples obtained at lower temperatures. The diffraction peaks also indicate that the carbon materials are found in a semicrystalline structure in these samples. A relatively high diffraction peak at 2 theta of approximately 26° was attributed to the (002) plane of the hexagonal graphite, suggesting a good graphitic structure of carbon. To calculate the CNC particles size, the Scherrer’s equation was applied [48]. The approximate particles size given by Scherrer’s calculation, based on the plane (002) of all of the prepared carbon material, is in the range of 8.2–8.8 nm, as summarized in Table 1. It can be observed that the carbon crystallite size increases as the temperature of pyrolysis increases.

**Figure 4:**
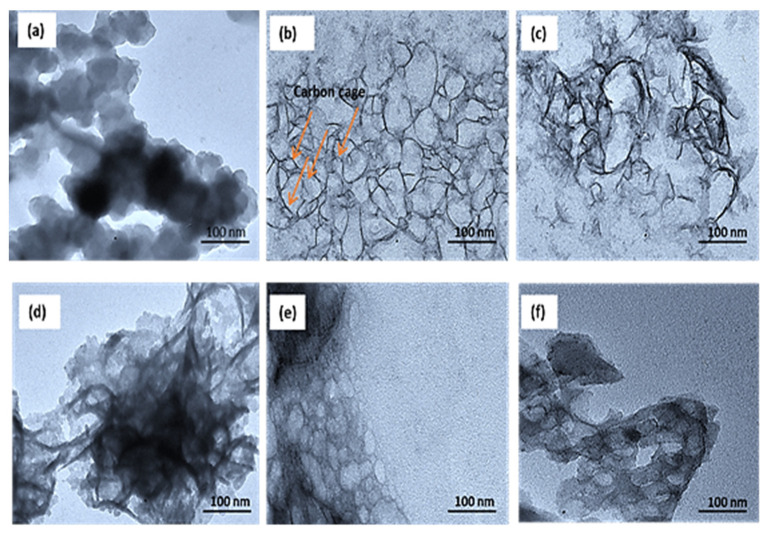
(**a**–**c**), TEM images of the carbon material prepared from pyrolysis of Ppy as the carbon source at 600 °C, 750 °C, and 900 °C, respectively. (**d**–**f**), TEM images of the carbon material prepared from pyrolysis of PANi as the carbon source at 600 °C, 750 °C, and 900 °C, respectively.

**Figure 5:**
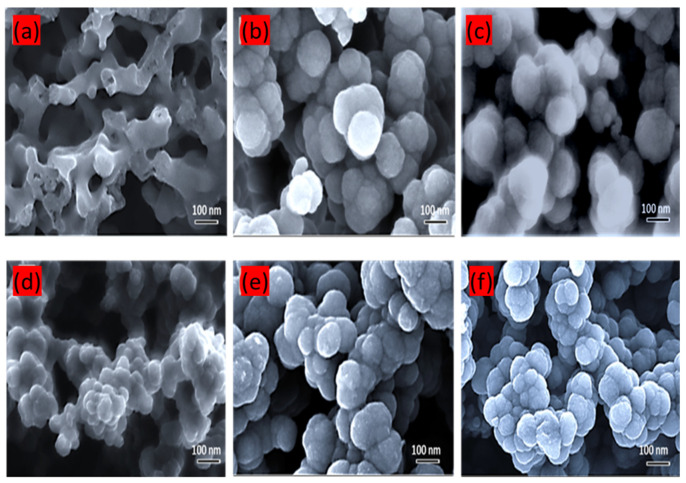
FESEM images for (**a**) CNC-Ppy600, (**b**) CNC-Ppy750, (**c**) CNC-Ppy900, (**d**) CNC-PANi600, (**e**) CNC-PANi750, and (**f**) CNC-PANi900.

**Figure 6:**
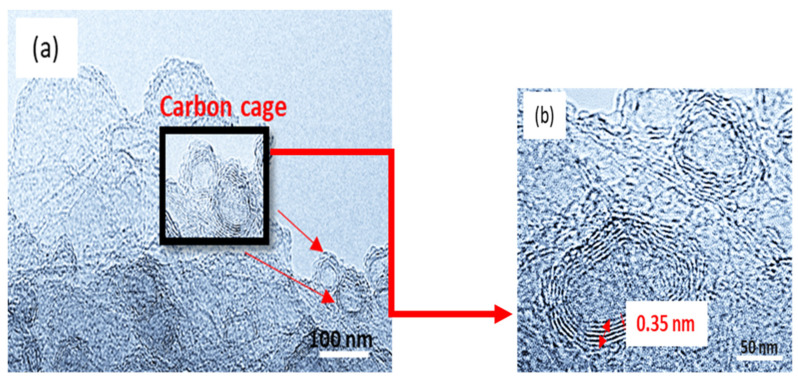
(**a**) HRTEM images of CNC at an operational temperature of 750 °C, showing the typical high-resolution TEM images of the shell for a carbon nanocage, and (**b**) close-up of the carbon cage image and it fringes.

To study the structural and morphology of prepared supports, TEM was carried out for both PANi and Ppy carbon sources, and the results are shown in Figure 4a–f. As mentioned earlier, all samples were prepared using the same pyrolysis method but with different sources and temperatures, as explained in the experimental section. The TEM images presented in Figure 4a,d show carbon materials obtained at the pyrolysis temperature of 600 °C for 2 h. It is evident that these carbon materials exhibit poor crystallinity and particle agglomeration is observed. On the other hand, Figure 4b,e show the successful formation of hollow nanocage carbons with a core–shell structure. These hollow CNCs possess graphitic shells and obtained after the removal of solid core using 1 M HNO_3_. The darker region in Figure 4d also reveals the agglomeration layer within the carbon particles. The average size of the obtained carbon particles is in range of 100–130 nm. This observation is in agreement with the SEM images in Figure 5. Various sizes of hollow CNCs, including particles with irregular spherical shape, can be observed in these samples (Figure 4b,e), which is consistent with the high-magnification images obtained by HRTEM and shown in Figure 6a,b. However, at an increased temperature of 900 °C, the carbon cage structure breaks down, as illustrated in Figure 4f, where TEM images indicate a significant fraction of broken carbon cages at the higher temperature.

Figure 5a–f show the SEM images of the as-synthesized carbon nanocages (CNC) obtained using the simple pyrolysis route. A close inspection reveals the spherical nanoparticles of the carbon material with the average diameter in the 100–130 nm range. The morphology of these prepared carbon nanocages does not exhibit any obvious differences when prepared at different temperatures and using different carbon sources. However, at a lower pyrolysis temperature for CNC-Ppy600 (Figure 5a), the shape of the carbon support is not clearly discernible. The carbon nanocages also display a moderate degree of agglomeration, which is consistent with the TEM images discussed earlier. In this carbon nanocage synthesis process, the pyrolysis of PANi and Ppy (as carbon sources) takes place on the surface of the MgO template particles. Subsequently, the carbon nanocages are obtained after the acid leaching process using 1 M HNO_3_, and the morphology of the metal template determines the shape or dimensions of the carbon nanocages. However, according to the SEM analysis, the CNCs synthesized at different temperatures exhibit the same spherical shape.

Figure 6 shows the further observations by high-resolution TEM (HRTEM) of CNC-PPy750 that revealed that it is composed of a multiwall structure of graphitic layers. The graphitic layers exhibit uniform thickness. The HRTEM image in Figure 6a shows that the spacing between neighboring lattice fringes is approximately 0.35 nm, corresponding to the (002) plane of the carbon cage lattice. The images also demonstrate that the CNC consists of a multiwall layer of graphitic carbon. An examination of Figure 6a shows that the thickness of the carbon wall is in the range of 1.5 to 3.5 nm owing to the moderate pyrolysis temperature (750 °C). The presence of a minor amount of amorphous material is also observed, but this was only found on the top surface of the prepared carbon nanocages. HRTEM-EDS of the CNC-PPy750 sample reveals its elemental composition, with 86.98% of C element, which is the highest among the prepared hollow CNCs, 6.72%. The sample also contains 6.72% of N element and 6.30% of O element in the CNC-Ppy750 after washing and drying process. The presence of N element is believed to originate from the N contained within the polypyrrole polymer ring. In catalysis or electrocatalysis, the addition or existence of the N element plays a crucial role in reducing or overcoming the agglomeration during the dispersion of catalyst on the carbon support [34]. Furthermore, it can enhance the catalytic activity in the methanol electrooxidation process [23,49]. This is attribute to the high affinity of N element, which converts the nonpolar covalent bonds of the carbon matrix to the polar bonds. Moreover, it creates anchoring sites that are advantageous for high loading of platinum catalyst.

The results for the porosity of the prepared carbon materials, calculated using the Barret–Joyner–Halenda (BJH) method from the desorption branch, are presented in Table 2. These results indicate that the carbon materials exhibit mesoporous characteristics. From nitrogen adsorption/desorption isotherm curve, a flat graph at relative low pressure (P/Po ≤ 0.6) is due to the absorption of micropore in the prepared sample. At a relatively higher-pressure region (0.6 < P/Po < 1.0), the graph indicates an increase in the adsorption capacity of the sample, which typically occurs in mesoporous materials due to the adsorption of monolayer and/or multilayer nitrogen molecules.

It is evident that the specific surface area (SSA) plays a crucial role in providing a larger number of active sites for platinum (Pt) deposition. Typically, the size of the synthesized Pt particle deposited on the support is greater than 3 nm. Theoretically, the micropore size is below 2 nm. Consequently, the Pt particles cannot enter the micropores with the size below 2 nm. Therefore, a carbon support with mesopore sizes is highly advantageous for Pt particle deposition.

**Figure 7:**
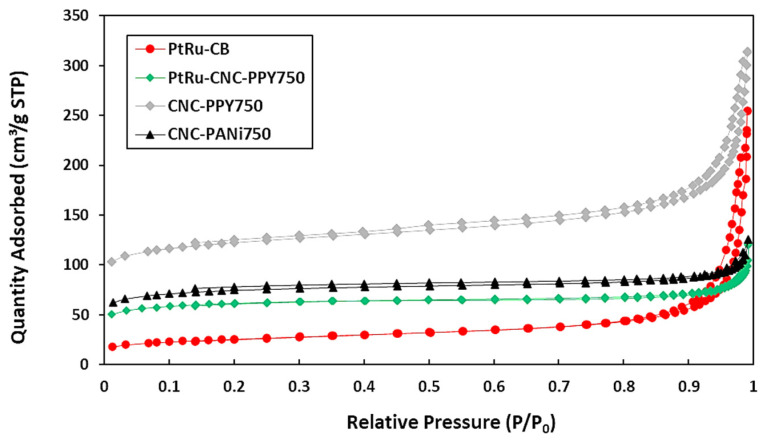
N_2_ adsorption-desorption isotherm for CNC-Ppy750, CNC-PANi750, PtRu/CNC-Ppy750, and PtRu/CB.

Figure 7 shows the N_2_-adsorption–desorption curve for N_2_ adsorption–desorption isotherm of CNC-Ppy750, CNC-PANi750, PtRu/CNC-Ppy750, and PtRu/CB. Based on the isotherm linear curves, all samples exhibit type IV isotherms (according to IU-PAC classification) with hysteresis H3, indicating the presence of a mesoporous structure in each sample. Additionally, this type of pore is beneficial in enhancing the catalyst homogeneity, improving stability of catalyst, and increasing the catalytic performance [50]. Among the samples, CNC-PPy750 demonstrates the highest specific surface area (SSA) of 416.10 m^2^g^−1^ with a corresponding pore size diameter of 39.2 nm. In comparison, CNC-PANi750 has an SSA of only 253.86 m^2^/g and a pore size diameter of 25.9 nm. As a result, CNC-PPy750 was selected as the support for PtRu catalyst doping, specifically PtRu/CNC-PP750. The high specific surface area is important factor for an excellent catalyst support. However, the SSA of PtRu/CNC-PP750 decreases after dispersion of PtRu metal on the CNC support. Nonetheless, the PtRu/CNC-PP750 electrocatalyst still shows a higher SSA and pore diameter size compared to the PtRu/CB, as presented in Table 2. The pore diameter for PtRu/CNC-Ppy750 and PtRu/CB is 2.8 nm and 3.1 nm, respectively. These values can be used to estimate the particle size. Smaller particle sizes have a higher surface-to-volume ratio, which benefits catalyst activity, solubility, and tends to alter the substance’s toxicity profile. The influence of SSA will be discussed further in relation to the catalytic activity of MOR in the electrochemical testing section.

**Figure 8:**
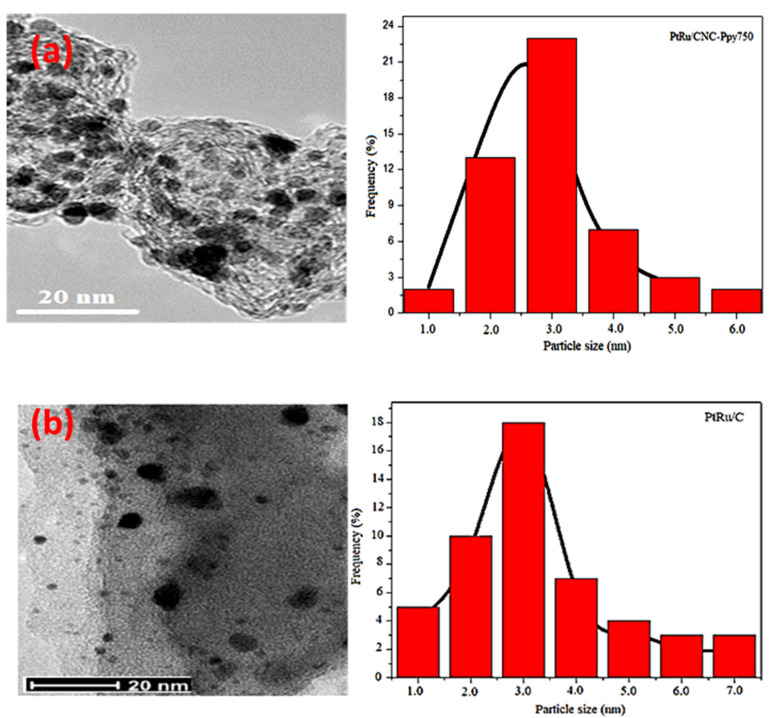
TEM images and histogram graph of particle size distribution for (**a**) PtRu/CNC-Ppy750 and (**b**) PtRu/CB electrocatalyst.

Figure 8 presents TEM images and particle size distribution for the prepared electrocatalyst, namely PtRu/CNC-PPy750 and PtRu/CB synthesized using the microwave-assisted reduction process. The TEM images reveals that the PtRu particles in PtRu/CNC-Ppy750 are in spherical shape and uniformly dispersed on CNC support with no agglomeration or separation from the supports. On the other hand, the PtRu particles that were dispersed on commercial carbon black support tend to aggregate, resulting in the formation of larger particles agglomerates measuring over 8 nm. Additionally, the PtRu particles observed on PtRu/CNC-Ppy750 are smaller ranging from 2.5 to 3.0 nm, which is smaller than the particle size range obtained in PtRu/CB, ranging from 2.8 to 3.4 nm. The uniform and homogeneous dispersion of PtRu particles on CNC can be attributed to the N element from Ppy source as detected by HRTEM-EDS. Meanwhile, the function of N element has been discussed previously by benefits in improve the particles dispersion onto support and increase catalytic activity. The role of the N element has been previously discussed, highlighting its benefits in improving particle dispersion onto the support and enhancing catalytic activity.

**Figure 9:**
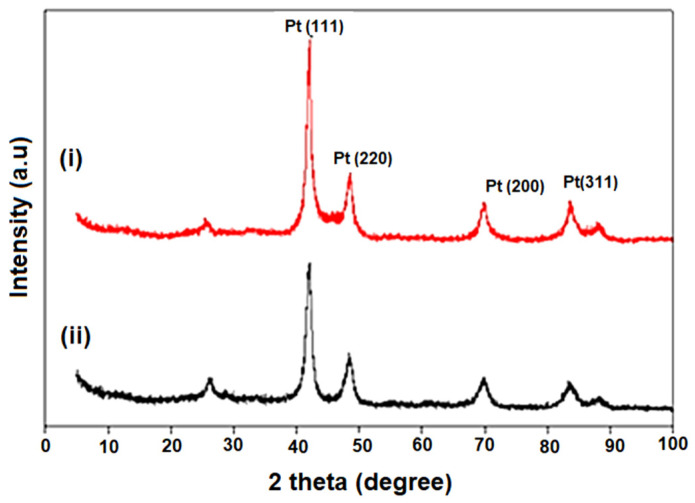
XRD analysis for (**i**) PtRu/CB and (**ii**) PtRu/CNC-Ppy750 electrocatalyst.

The XRD pattern (Figure 9) confirms that Pt can be indexed to the face-centered cubic (fcc) structure [51]. As expected, all peaks are shifted to the higher 2 theta due to the interaction of second metal, Ru with Pt. Furthermore, no peaks of Ru were detected on XRD analysis, indicating the successful formation of bimetallic PtRu on the electrocatalyst system [52]. Additionally, the XRD pattern the diffraction peaks of PtRu/CNC-Ppy750 are shorter and broader compared to PtRu/CB. This can be attributed to the smaller crystallite size of PtRu particles (3.2 nm) in the PtRu/CNC-Ppy750 sample, in contrast to PtRu/CB (3.5 nm). This finding is consistent with results obtained from TEM, indicating that PtRu/CNC-Ppy750 demonstrated the smaller particle size than PtRu/CB. The higher performance of PtRu/CNC-Ppy750 in MOR can also be attributed to the smaller particle size, as calculated using Scherrer’s equation based on plane (111) The smaller crystallite particle size can also be used to estimate the particle size of PtRu nanoparticles. Moreover, the smaller PtRu particle size and uniform dispersion of PtRu particles on the CNCs play a crucial role in enhancing MOR. Therefore, the CNCs support can be serve as an ideal support carbon for dispersing of PtRu nanoparticles, making it a promising electrocatalyst for MOR and DMFC applications.

**Figure 10:**
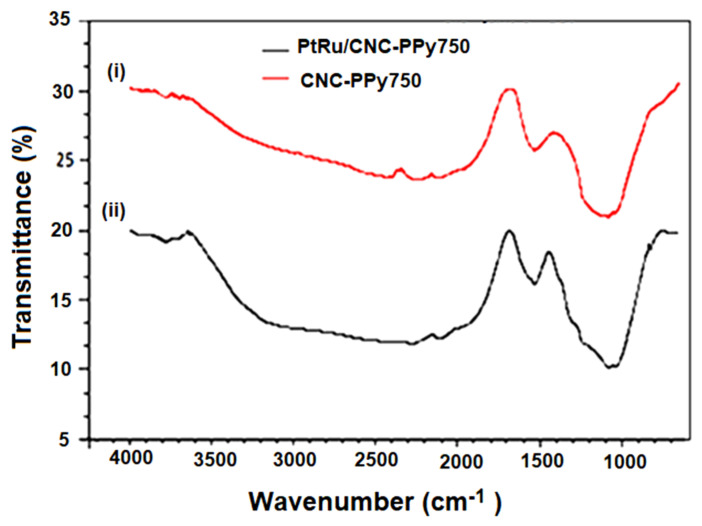
FTIR spectrum for (**i**) CNC−PPy750 support and (**ii**) PtRu supported CNC−PPy750 at the pyrolysis temperature of 750 °C.

FTIR spectra presented in Figure 10 confirms the presence of functional groups in PtRu/CNC-Ppy750. The FTIR spectra were recorded in the range from 650 to 4000 cm^−1^ After doping with the PtRu catalyst (PtRu/CNC-Ppy750), the peaks appearing in the spectra do not show significant differences compared to the spectra of pure CNCs. Only the peak at approximately 800 cm^−1^ appears after doping with the PtRu catalyst, can be attributed to the presence of PtRu metal in the prepared samples. The peaks observed at approximately 3300–1600 cm^−1^ indicate the O-H stretching and vibrations of water molecules on the surface of the prepared samples [46]. Peaks at approximately 1530 cm^−1^ correspond to quinoid rings or the C-C benzene ring, although this peak can also be attributed to the stretching of C=O groups from ketone or carbonyl functional groups [44]. The peaks at approximately 1225 cm^−1^ and ~1050 cm^−1^ correspond to the stretching of aliphatic amines (C-N) and alkoxy groups (C-O), respectively. These results confirm the existence of functional groups on the CNC support and PtRu/CNC-Ppy750 electrocatalyst.

### 3.2. Electrochemical Performance of PtRu/CNC-Ppy750 Electrocatalyst

**Figure 11:**
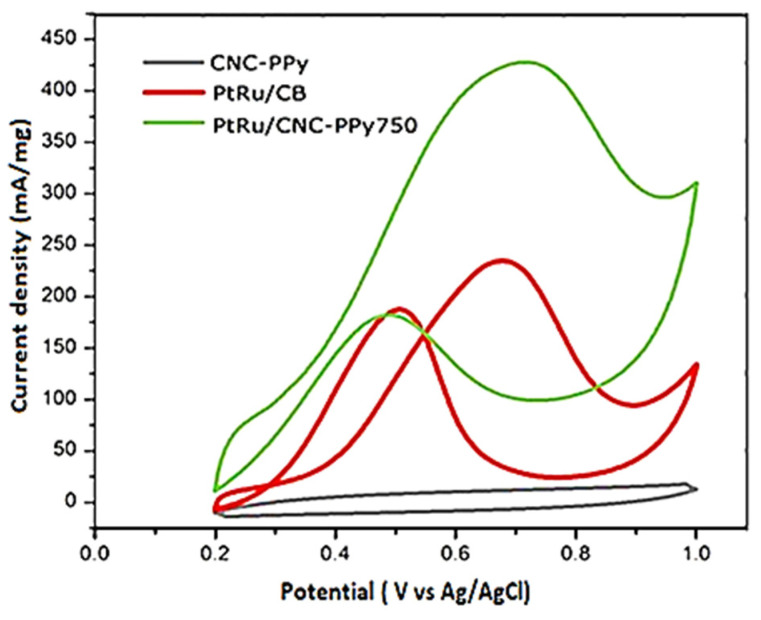
Cyclic voltammetry (CV) analysis for the PtRu/CNC-Ppy750 and PtRu/CB electrocatalysts in 0.5 M H_2_SO_4_ and 2.0 M CH_3_OH solution at a scan rate of 50 mV s^−1^.

The electrochemical activity and stability of the synthesized catalyst were examined through cyclic voltammetry (CV) and chronoamperometry (CA) in the methanol oxidation reaction (MOR). An important parameter used to evaluate the electrocatalytic performance is the ratio of current densities associated with the anodic peaks in the forward (I_f_) and reverse (I_b_) directions [53]. The larger I_f_/I_b_ value of the PtRu/CNC-Ppy750 sample (Table 3) suggested good oxidation of methanol to CO during the anodic scan. The experimental results provided in Table 3 clearly demonstrate that the prepared PtRu/CNC-Ppy750 exhibits a higher I_f_/I_b_ value compared to PtRu/CB, indicating an improvement in CO tolerance. Therefore, CO can be more efficiently oxidized to carbon dioxide for the PtRu/CNC-Ppy750 electrocatalyst compared to PtRu/CB. The current density values measured for the Pt catalyst alone at a potential of 0.715 V, when deposited on the graphitic CNCs-PPy material, show a higher forward peak of 427 mA/mg^−1^ compared to that on commercial carbon black (248 mA/mg^−1^). As observed in the graph (Figure 11), the CNC-PPy750 alone does not exhibit any catalytic activity. However, after doping the Pt and Ru catalyst onto this support, a remarkable catalytic activity towards MOR is observed. The onset potential for PtRu/CNC-Ppy750 is shifted to a higher potential value (0.71 V), which is higher than that of commercial PtRu/C (0.68 V). This indicates that the catalytic activity of PtRu/CB is faster than that of our PtRu/CNC-Ppy750. Despite the slightly higher onset potential observed for PtRu/CNC-Ppy750, it still demonstrates superior MOR performance. This could possibly be attributed to an increased OH coverage on the Pt surface [54].

Given the bifunctional mechanism of the PtRu catalyst in the methanol electrooxidation reaction, the mechanism can be represented by the following reactions [11]:

Pt + CH_3_OH → Pt–(CH_3_OH)_ads_ → Pt–CO_ads_ + 4H^+^ + 4e^−^ (3)

Ru + H_2_O → Ru–(H_2_O)_ads_ → Ru–OH_ads_ + H^+^ + e^−^ (4)

Pt–CO_ads_ + Ru–OH_ads_ → Pt + Ru + CO_2_ + H^+^ + e^−^ (5)

According to the above reaction, in the first step, methanol is adsorbed on Pt sites and undergoes a dehydrogenation reaction (reaction 3), leading to the production of CO_ads_. In a parallel reaction step, water molecules dissociate on Ru sites, generating OH species (reaction 4). The inductive behavior as mentioned above is determined to happen in reactions with absorbed species, the coverage of which changes with potential [55]. The oxidation of weak CO_ads_ species begins as the potential increases, creating active Pt sites on the adsorbed carbon monoxide layers for the subsequent adsorption and oxidation of CH_3_OH [56]. As a result, the rate of methanol electrooxidation experiences a sudden increase. This behavior is known as “pseudoinductive” and arises from the “relaxation phenomenon” between the adsorption/dehydrogenation of methanol and the oxidation/adsorption of CO-like species [57].

Table 4 shows the performance comparison of PtRu-based catalysts on various carbon support materials in the methanol electrooxidation reaction. This study has demonstrated that the PtRu catalyst supported on CNC-PPy (PtRu/CNC-Ppy750) used in this study exhibits the highest catalytic activity compared to previous works.

As shown in Table 3, the electrochemical active surface area (ECSA) for PtRu/CNC-Ppy750, and PtRu/CB electrocatalyst was calculated to be 16.0 m^2^g^−1^ and 14.06 m^2^g^−1^, respectively using same formula applied by Sebastian and co-worker, as follows [65]:

ECSA (m^2^g ^−1^ _Pt_) = Q/Γ·W_Pt_ (6)

Referring to the above formula (ECSA), Q represents the charge density or the area beneath the experimental cyclic voltammetry (CV) graph. Γ (2.1 Cm_Pt_^−2^) is a constant denote as the charge necessary for reducing a monolayer of protons on the Pt surface, and W_Pt_ refers to the loading of Pt (g_Pt_) on the electrode. The PtRu/CNC-Ppy750 possesses a higher ECSA compared to PtRu/CB electrocatalysts, possibly due to its smaller particle size and crystallite size of PtRu nanoparticles on the CNCs support. The estimated crystallite size for PtRu in PtRu/CNC-PPy750 is smaller and has a higher ECSA value compared to PtRu/CB. The catalyst and reaction surface area can be enhanced by the smaller crystallite size. Moreover, the trend in crystallite size is paralleled by the trend in ECSA value for PtRu/CNC-PPy750 and PtRu/CB. This result also suggests that the PtRu/CNC-PPy750 is more electrochemically accessible, which is vital for the MOR [66,67].

**Figure 12:**
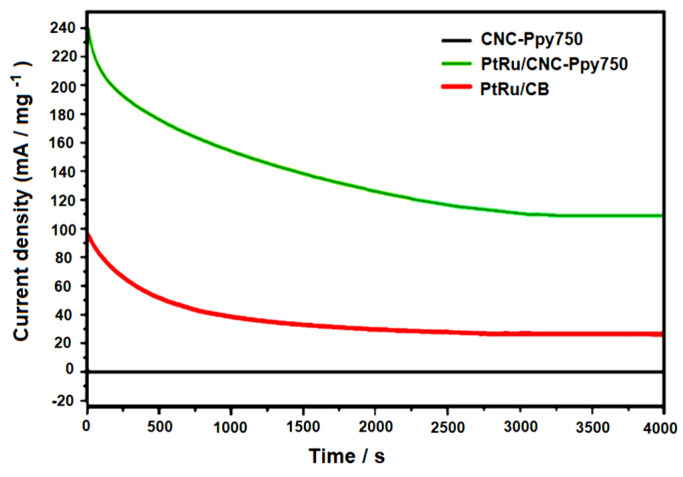
Representative chronoamperometry (CA) curves of the synthesized PtRu/CNC−PPy750 and PtRu/CB catalysts obtained in a 2.0 M methanol solution + 0.5 M sulfuric acid at a scan rate of 50 mV s^−1^ and nitrogen gas flow.

Figure 12 displays the results of the CA measurements conducted to provide further additional information regarding the electrocatalysts’ stability towards methanol oxidation. The CA curves of CNC, PtRu/CNC-Ppy750 and PtRu/CB were recorded at a constant potential of 0.4 V for 4000 s. The oxidation current continuously decreased for both electrocatalysts (PtRu/CB and PtRu/CNC-PPy750) from 0 to 4000 s. This observation is likely due to the formation of several intermediate species during methanol oxidation, such as Co_ads_, CH_3_OH_ads_, and CHO_ads_ [68]. A rapid decay also occurred in the first 500 s for both electrocatalysts, resulting from the presence of CO intermediate molecules during the methanol oxidation reaction [56]. However, after approximately 3000 s of CA testing, the PtRu/CNC-PPy750 electrocatalyst exhibited a higher initial and significantly higher final current response than the PtRu/CB electrocatalyst (i.e., approximately 113.8 mA mg _Pt_^−1^ for PtRu/CNC-Ppy750 and only 30.5 mA mg _Pt_^−1^ for PtRu/CB). Moreover, the current density decay rate was slower for PtRu/CNC-Ppy750 than for PtRu on carbon black support, indicating that the PtRu/CNC-Ppy750 electrocatalyst demonstrated better stability in methanol oxidation compared to PtRu/CB. These findings suggest that the Pt and Ru particles deposited on graphitic CNCs support are significantly more active, albeit with slower stability, than the PtRu/CB commercial carbon support in the methanol oxidation reaction. Additionally, the slower decay observed during the first 500 s indicates that CNC is a more stable carbon support material for PtRu particles than commercial carbon black at the same PtRu loading.

Table 5 represents the retention values obtained from CA analysis. The PtRu/CNC electrocatalyst achieved the highest retention rate of 47.12% and exhibited a slower decrease in current density during the electrooxidation of methanol. Even after 3000 s, this electrocatalyst maintained high stability, with a current density value of 113.8 mA/mg. These results also indicate that the CNC support has a low corrosion rate, which is a crucial property for a catalyst support material in fuel cell applications [19].

From all the results, it is observed that the PtRu/CNC-Ppy750 electrocatalyst exhibits significantly improved electrocatalytic activity and high stability compared to PtRu on commercial carbon black. This study attributes the substantial improvement to several factors. Firstly, CNC with its unique hollow structure, relatively high surface area, and high pore volume of CNC-PPy750 are beneficial for catalyst dispersion and excellent properties as a catalyst support. Secondly, the deposition of PtRu particles on the graphitic structure of CNC support with unique properties may contribute to better electrical conductivity due to the homogeneous and uniform dispersion of PtRu. The uniform dispersion with small particle size of PtRu (as observed by TEM) on the large surface area of CNC allows for uniform catalyst ink preparation and maximizes the activity of this electrocatalyst during methanol electrooxidation (resulting in a large ECSA value). Thirdly, the N content in the CNC support helps overcome the aggregation and agglomeration of catalyst particles during electrocatalyst preparation, further enhancing the electrooxidation performance.

### 3.3. DMFC Performance of PtRu/CNC-Ppy750 Electrocatalyst

**Figure 13:**
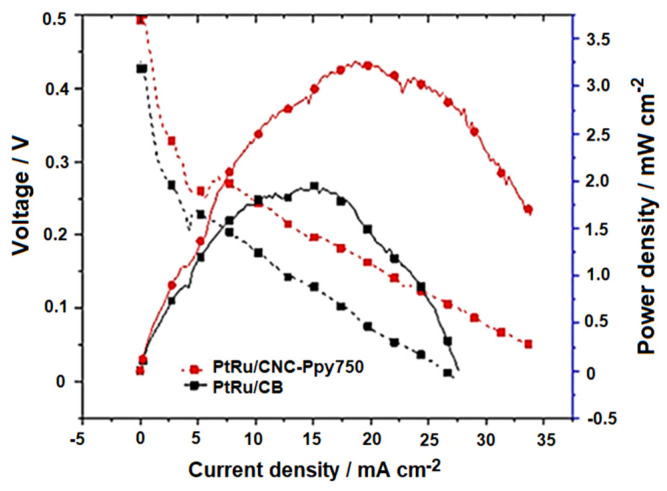
Polarization curves (current-voltage) for DMFCs with PtRu/CNC−Ppy750 and PtRu/CB using 2 mg cm^−2^ catalyst loading in 2.0 M methanol solution at room temperature.

Figure 13 presents the current-voltage (IV) polarization curve for PtRu/CNC-Ppy750 and commercial PtRu/CB electrocatalysts. From the DMFC single-cell tests, it is observed that the PtRu/CNC-Ppy750 electrocatalyst exhibits significantly higher potential performance, around 1.7 times higher than that of the commercial PtRu/CB. The maximum power density achieved by the PtRu/CNC-Ppy750 electrocatalyst is 3.35 mWcm^−2^, while that of PtRu/CB is 1.95 mWcm^−2^. These results confirm the superior performance of PtRu/CNC-Ppy750 when compared to previous studies utilizing commercial PtRu/C electrocatalysts in DMFC passive mode systems, as outlined in Table 6. The overall performance obtained from the electrochemical and single cell measurements leads to the conclusion that the combination of PtRu bimetallic catalysts with nanostructured carbon (CNC) holds great potential and represents an ideal candidate for replacing PtRu/C in DMFC technology.

**Figure 14:**
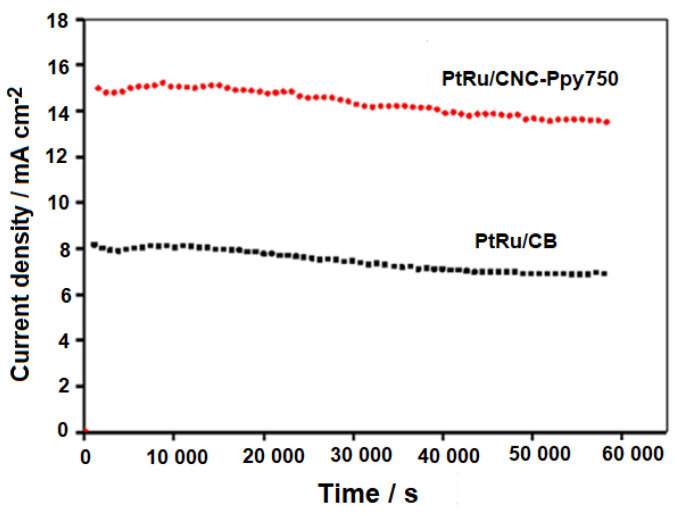
Plot of the current density at the constant cell voltage 0.2 V against time for DMFCs with PtRu/CNC-Ppy750 and PtRu/CB measured in a 2.0 M methanol solution.

Figure 14 illustrates the long-term stability of PtRu/CNC-Ppy750 and PtRu/CB electrocatalysts, represented by the plot of current density at a constant cell voltage of 0.2 V against time for DMFCs tested in a 2.0 M methanol solution at room temperature (298.15 K). The DMFC test with PtRu/CNC-Ppy750 electrocatalyst exhibited a significantly higher current density. The obtained value was almost two times higher than that of the PtRu/CB with commercial carbon support and was maintained throughout the 60,000 s of DMFC testing. Overall, the experiments conducted in this study consistently demonstrated that the PtRu/CNC-Ppy750 electrocatalyst exhibits superior and higher activity in both methanol oxidation reaction (MOR) and DMFC performance compared to the PtRu/CB with commercial carbon black support.

## 4. Conclusions

In summary, hollow graphitic CNC from polypyrrole source namely, CNC-Ppy750 with a unique structure, highly porous structure, and high surface area (416 m^2^/g) was successfully synthesized through polymerization followed by pyrolysis. The CNC-PPy750 was then doped with PtRu catalyst using a chemical reduction method and microwave-assisted technique. Electrochemical tests revealed that PtRu/CNC-Ppy750 exhibited higher current density (427 mA mg^−1^) compared to PtRu on commercial carbon black (248 mA mg^−1^). Additionally, PtRu/CNC-Ppy750 demonstrated a higher ECSA value and greater CO-tolerance, as evidenced by the calculation of I_f_/I_b_ (yielded a value of 2.34. I_f_/I_b_). The improved methanol oxidation reaction and electrocatalytic activity of PtRu/CNC-Ppy750 can be attributed to the unique structure of the CNC support, which possesses a high surface area and nitrogen content, facilitating the high dispersion of PtRu nanoparticles on the CNCs. Furthermore, the smaller particle size of PtRu (confirmed by XRD and TEM analysis) and the uniform dispersion of PtRu particles on CNCs contribute to the higher ECSA value in methanol oxidation (16.23 m^2^/g). Finally, in terms of DMFC performance, PtRu/CNC-Ppy750 exhibited superior performance with a power density of 3.35 mW cm^−2^ compared to PtRu on commercial carbon support (PtRu/CB).

## Figures and Tables

**Table 1:** Estimated crystallite size of carbon materials obtained from XRD analysis (based on plane 002).

Support Name	Temperature of Pyrolysis (°C)	Est. Crystallite Size (nm)
CNC-PANI600	600	8.2
CNC-PANI750	750	8.3
CNC-PANI900	900	8.8
CNC-Ppy600	600	8.2
CNC-Ppy750	750	8.2
CNC-Ppy900	900	8.3

**Table 2:** BET analysis for the samples’ porosity with different temperatures.

Samples	CNC-PANI750	CNC-PPy750	PtRu/CNC-Ppy750	PtRu/CB
S_BET_ (m^2^g^−1^)	253.86	416.10	210.15	89.62
V_Total_ (cm^3^g^−1^)	0.16	0.41	0.06	0.12
S_mesopore_ (m^2^g^−1^)	74.02	131.10	65.39	61.02
S_Micropore (_m^2^g^−1^)	179.84	285.00	144.76	28.60
D_pore_ (nm)	25.9	39.2	2.8	3.1

S_BET_ = BET specific surface area, V_Total_ = Total pore volume at P/Po = 0.99, S_mesopore_ = mesopore surface area, S_micropore_ = micropore surface area, D_pore_ = pore diameter.

**Table 3:** Electrocatalytic activity towards the methanol oxidation reaction (MOR) characteristics of the PtRu/CNC-Ppy750 and PtRu/CB electrocatalysts.

Sample	Onset Potential (V vs. Ag/AgCl)	Peak Potential (V vs. Ag/AgCl)	ECSA (m^2^g^−1^)	Current Density (mA mg^−1^)	CO Tolerance (I_f_/I_b_)
PtRu/CNC-PPy750	0.325	0.71	16.23	427	2.34
PtRu/CB	0.391	0.68	14.06	248	1.25

**Table 4:** Performance comparison of PtRu-based catalyst on different carbon support materials in the methanol oxidation reaction (MOR) by previous researchers.

Catalysts	Catalyst Loading (wt. %)	Scan Rate (mVs^−1^)	Current Density (mAmg^−1^)	Reference
PtRu/CNC-PPy750	20	25	427	this research
PtRu/CB	20	25	248	this research
PtRu/CNF	20	20	186.29	Abdullah et al., 2018 [58]
PtRuFeNi/MWCNT	20	50	31	Basri et al., 2014 [59]
PtRu-TNT	10	50	3.3 mA/cm^2^	Abdullah et al., 2016 [46]
PtRu/HollowCarbon Sphere	-	50	323.6	Liu et al., 2013 [56]
PtRu/CeCNF	15	20	290	Kunitomo et al., 2016 [60]
PtRu/CNT	40	50	335	Cheng et al., 2014 [61]
Pt/Graphene sheet	-	20	202.2	Zhao et al., 2014 [62]
PtRu/Graphene	40	50	200	Woo et al., 2013 [63]
PtRu/MWCNT	-	50	~24	Chen et al., 2017 [64]

**Table 5:** Long term stability data of prepared electrocatalysts obtained from CV analysis.

Electrocatalysts	j*_i_* (mA/mg)	j*_f_* (mA/mg)	Retention Value (%)
PtRu/CNC-PPy750	241.5	113.8	47.12
PtRu/CB	98.0	30.5	31.12

j*_i_* = Initial current density, j*_f_* = Final current density.

**Table 6:** Comparison of the DMFC performance in the passive mode with the results obtained by other researchers.

Electrocatalysts	Electrocatalyst Loading (mg/cm^2^)	Power Density (mW/cm^2^)	Reference
PtRu/CNC-Ppy750	2	3.35	This study
PtRu/CB	2	1.95	This study
PtRu/C	2	2.20	Abdullah et al., 2018 [58]
PtRu/C	2	1.7	Hashim et al., 2009 [69]
PtRu/C	2	3.0	Shimizu et al., 2004 [70]

## Data Availability

Data are available within the article.
